# The efficacy of mirabegron additional therapy for lower urinary tract symptoms after treatment with α1-adrenergic receptor blocker monotherapy: prospective analysis of elderly men

**DOI:** 10.1186/s12894-016-0165-3

**Published:** 2016-07-29

**Authors:** Tomohiro Matsuo, Yasuyoshi Miyata, Katsura Kakoki, Miki Yuzuriha, Akihiro Asai, Kojiro Ohba, Hideki Sakai

**Affiliations:** Department of Urology, Nagasaki University Graduate School of Biochemical Sciences, 1-7-1 Sakamoto, Nagasaki, 852-8501 Japan

**Keywords:** Mirabegron, Elderly male, Overactive bladder, α1-adrenergic blockers

## Abstract

**Background:**

Mirabegron is a β3-adrenoreceptor agonist developed for treatment of overactive bladder (OAB). α1-Adrenergic receptor blockers are effective for lower urinary tract symptoms (LUTS) in male patients. However, the efficacy of mirabegron additional treatment in elderly male patients with persistent male LUTS, especially in OAB after monotherapy with α1-adrenergic blockers, is not fully understood.

**Methods:**

This study was conducted in male LUTS patients who were ≥ 65 years of age and had persistent OAB symptoms, regardless of whether they took an α1-adrenergic receptor blocker orally. Before and 12 weeks after mirabegron additional therapy (50 mg once daily), we evaluated the efficacy of this treatment using the Overactive Bladder Symptom Score (OABSS) and International Prostate Symptom Score (IPSS), and changes in the maximum flow rate (Qmax) and post-void residual urine volume (PVR). We evaluated patients overall and divided into two groups by age: young-old (from 65 to 74 years old) and old-old (from 75 to 84 years old).

**Results:**

Fifty men were enrolled in this study. Mirabegron additional therapy improved the total OABSS, total IPSS, and IPSS-quality of life (QOL) score. The voided volume (VV) and Qmax improved after treatment in patients overall. However, there was no significant change in PVR. The total OABSS, total IPSS, and IPSS-QOL score significantly improved in both of the young-old and old-old groups. However, a significant increasing of VV was detected in the young-old group. There were no significant differences in the Qmax or PVR in either group.

**Conclusions:**

Mirabegron additional therapy was effective for male patients whose persistent LUTS and particularly OAB was not controlled with α1-adrenergic receptor blocker monotherapy, and mirabegron did not have negative effects on voiding function. Additionally, mirabegron additional therapy was considered effective regardless of patient age.

**Trial registration:**

Trial registration number (TRN) trial registration number (TRN) and date of registration: ISRCTN16759097 in July 8, 2016.

## Background

Overactive bladder (OAB) is defined as a condition with characteristic symptoms of urinary urgency that is usually accompanied by frequency and nocturia, with or without urgency incontinence [1]. It is particularly burdensome to older people because of its higher prevalence, and because the impact of its symptoms may be more pronounced due to the increased burden of chronic comorbidities [2, 3]. The symptoms caused by OAB decrease patients’ quality of life (QOL), and OAB can lead to various pathological conditions such as increases in the fracture rate, sleep disturbances, and depressive feelings) [4, 5]. Anti-muscarinic agents are often used as first-line therapy for patients with OAB. However, unfortunately, about a third quarter of patients cannot continue taking these drugs due to unsatisfactory efficacy and various adverse events [6, 7].

In addition, mainly due to the adverse effects of anti-muscarinic drugs, Japanese clinical guidelines for both male lower urinary tract symptoms (LUTS) and benign prostatic hyperplasia recommend physicians to use α1-adrenergic blockers as the first choice drug for male LUTS patients regardless of the presence or absence of OAB symptoms [8, 9].

Mirabegron is a β3-adrenoceptor agonist approved for treating OAB in Europe, the United States, Canada, Japan, and Australia [10]. Mirabegron is a specific agonist, acting on β3-adrenoceptors in the human detrusor, the stimulation of which leads to active relaxation of the human detrusor in the storage phase, which increases bladder capacity without exerting an effect on voiding [11]. The efficacy and safety of mirabegron have been studied in several randomized trials. For example, SCORPIO, a large, randomized, placebo-controlled phase III study, evaluated the efficacy, safety, and tolerability of mirabegron over 12 weeks in patients with OAB [12], and TAURUS, a 1-year, randomized, double-blind, safety study, evaluated the safety, tolerability, and efficacy of mirabegron [13]. Both studies mainly improved storage symptoms, including urgency incontinence and increased voiding volume, and demonstrated that the efficacy of mirabegron lasted 4 weeks later and continued until the end of the observation periods. Furthermore, the rate of adverse events due to 50 mg mirabegron was similar to that of a placebo, and significantly lower than that of the anti-muscarinic drug tolterodine (4 mg) [12, 13]. In addition, other researchers have reported that mirabegron add-on therapy combined with α1-adrenergic receptor blockers is very effective for storage symptoms, even in male patients with OAB [14–16]. However, no studies have focused on elderly male patients aged 65 years old and over.

To our knowledge, the present study is the first to focus on the efficacy of mirabegron additional therapy in elderly male patients with OAB after treatment with α1-adrenergic receptor blocker monotherapy.

## Methods

This prospective study was conducted in male patients who were 65 years or older in age, had persistent LUTS and particularly OAB symptoms, and had been taking a regular dose of α1-adrenergic receptor blockers for more than 12 weeks. OAB was diagnosed using the Overactive Bladder Symptom Score (OABSS), and persistent OAB symptoms were defined as a total OABSS of 3 or more points with urinary urgency at least once per week [17]. Exclusion criteria were a post-void urine volume (PVR) of 50 mL, history of urinary retention, prior diagnosis of neurogenic bladder, urethral stricture, severe hypertension (systolic blood pressure ≥ 180 mmHg and/or diastolic blood pressure ≥ 110 mmHg) not well controlled by medication, renal insufficiency (glomerular filtration rate < 30 mL/min/1.73 m^2^), liver impairment, intention to have a child, urological malignancy, patients taking any anti-muscarinic drugs, or those considered unsuitable for the trial by the treating physicians.

The patients continued all of their prescribed drugs during this study period. Before and 12 weeks after mirabegron (Betanis®, Astellas Pharma Inc., Tokyo, Japan; 50 mg once daily) treatment was added to a previous α1-adrenergic receptor blocker for urinary symptoms, we evaluated the efficacy of the treatment using the OABSS and International Prostate Symptom Score (IPSS) to assess subjective symptoms, and we used uroflowmetry and PVR to assess objective symptoms. We measured the maximum flow rate (Qmax) using the Duet® Logic G2 system (Mediwatch UK Ltd., Rugby, UK) on free uroflowmetry and PVR using transabdominal ultrasound sonography (HI VISION Avius®, Hitachi-Aloka Medical, Ltd, Tokyo, Japan). Moreover, before mirabegron add-on treatment was administered, we evaluated the prostate volume (PV) using transabdominal ultrasound sonography.

The primary endpoint was the change in total OABSS. The secondary endpoints evaluated were the change in the subscale scores of the OABSS, total IPSS, each subscale score of the IPSS and IPSS-QOL, voided volume (VV) on free uroflowmetry, Qmax, and PVR. In this study, we compared these subjective and objective parameters between two groups defined according to the patients’ age: young-old (from 65 to 74 years old) and old-old (from 75 to 84 years old).

During the clinical study, the current α1-adrenergic receptor blocker that the patients had been taking orally was not changed to a different one. Additionally, no patients were taking multiple α1-adrenergic receptor blockers. The safety assessment included a change in adverse events. We observed patients’ complaints of adverse effects, and information on the adverse events was recorded throughout the study period.

All statistical analyses were performed using computer software (JMP 10; SAS Institute Inc., Cary, NC, USA). Differences in the changes in patients’ parameters from baseline to 12 weeks were examined using the Wilcoxon signed-rank test. *P* < 0.05 was considered statistically significant.

## Results

Fifty men were enrolled in this study. As shown in Table 1, overall, the mean ± standard deviation (SD) of patients’ age was 75.7 ± 7.6 years, and the mean ± SD PV was 33.7 ± 8.6 mL. All patients had taken a previous α1-adrenergic receptor blocker, including silodosin (26 patients), naftopidil (15), tamsulosin (8), and urapidil (1). Among the 50 patents, 22 (44.0 %) and 28 (56.0 %) were classified into the young-old group and old-old group, respectively. No other demographic or clinical parameters significantly differed between the young-old and old-old groups.

**Table 1:** Patients’ characteristics

	Overall	Young-old group	Old-old group	*P* value^a^
Number of patients (*N*/%)	50	22 (44.0)	28 (56.0)	-
Age (years)	75.7 ± 7.6	68.7 (2.7)	81.1 (5.3)	< 0.001
Prostate volume (mL)	33.7 ± 8.6	33.5 (7.6)	33.7 (9.5)	0.961
α1-adrenergic receptor blocker				
Silodosin (*N*/%)	26 (52.0)	13 (59.1)	13 (46.4)	
Naftopidil	15 (30.0)	5 (22.7)	10 (35.7)	
Tamsulosin	8 (16.0)	3 (13.6)	5 (17.9)	
Urapidil	1 (2.0)	1 (4.5)	0 (0)	

Data are shown as mean ± standard deviation

^a^Difference between the young-old group and old-old group

**Fig. 1:**
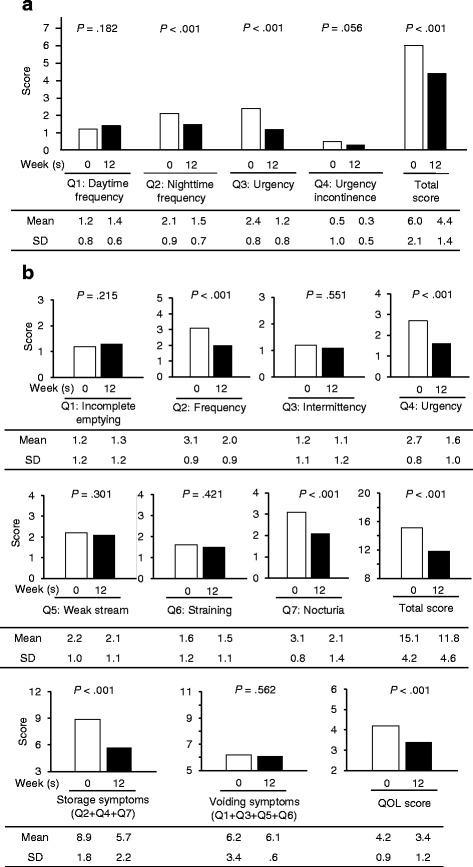
Changes in subjective symptoms. **a** shows changes in the Overactive Bladder Symptom Score (OABSS). Combination therapy with α-1 adrenergic receptor blocker and mirabegron for 12 weeks significantly improved the total OABSS, nighttime frequency (Q2), and urgency (Q3). **b** shows the change in the International Prostate Symptom Score (IPSS). Combination therapy significantly improved the IPSS total score, IPSS-quality of life, and storage symptoms (frequency [Q2], urgency [Q4], nocturia [Q7], and IPSS-storage symptoms [Q2 + Q4 + Q7]). However, combination therapy did not affect voiding symptoms (incomplete emptying [Q1], intermittency [Q3], weak stream [Q5], straining [Q6], and IPSS-voiding symptoms [Q1 + Q3 + Q5 + Q6]). The *white columns* show the scores at 0 weeks, and the *black columns* show scores at 12 weeks. SD, standard deviation

Figure 1a shows the change in the OABSS before and after mirabegron add-on treatment was administered to patients overall. Mirabegron add-on treatment improved the total OABSS (from 6.0 ± 2.1 to 4.4 ± 1.4, *P* < 0.001), and the nighttime frequency (OABSS Q2, *P* < 0.001) and urgency (OABSS Q3, *P* < 0.001) subscale scores of the OABSS after the 12-week treatment period. Although the urgency incontinence subscale score (OABSS Q4) improved after 12 weeks compared to that before treatment, the difference did not reach statistical significance (*P* < 0.05). Similarly, mirabegron add-on treatment significantly improved the total IPSS (before 15.1 ± 4.2 to 11.8 ± 4.6, *P* < 0.001); frequency (IPSS Q2, *P* < 0.001), urgency (IPSS Q4, *P* < 0.001), and nocturia subscales (IPSS Q7, *P* < 0.001); and IPSS-QOL score (*P* < 0.001) after treatment (Fig. 1b). There were no significant differences in voiding symptoms, including incomplete emptying, intermittency, weak stream, and straining before and after 12-week mirabegron add-on treatment.

**Fig. 2:**
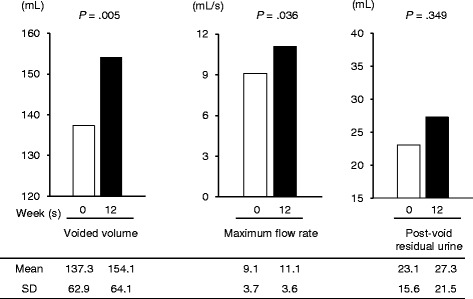
Changes in the measurements of objective symptoms in patients overall. The *white columns* show the values at 0 weeks, and the *black columns* show those at 12 weeks. After 12 weeks of α1-adrenergic receptor blocker and mirabegron combination therapy, the voided volume and maximum flow rate improved significantly. Post-void residual urine increased at the end of the period; however, this change was not statistically significant. SD, standard deviation

In the entire patient, changes in the objective parameters of VV, Qmax, and PVR are shown in Fig. 2. VV on free uroflowmetry increased from 137.3 ± 62.9 mL to 154.1 ± 64.1 mL after treatment (*P* = 0.005). Similarly, Qmax improved from 9.1 ± 3.7 mL/s to 11.1 ± 3.6 mL/s (*P* = 0.036). However, no significant change was detected in PVR (from 23.1 ± 15.6 mL to 27.3 ± 21.5 mL, *P* = 0.349).

Next, changes of these parameters on subjective symptoms and objective measurements depending on age (young-old and old-old group) were showed in Table 2. The total OABSS and nighttime frequency (OABSS Q2), and urgency (OABSS Q3) subscales were improved in both groups. However, urgency incontinence (OABSS Q4) was improved only in the young-old group (*P* = *0.021*; old-old group, *P* = *0.686*). Significant improvements were also observed in the total IPSS; IPSS-QOL; and frequency (IPSS Q2), urgency (IPSS Q4), and nocturia (IPSS Q7) subscales in both groups. No IPSS-related parameter significantly differed between the young-old group and the old-old group. However, although all IPSS parameters were unchanged or decreased in the young-old group after treatment, incomplete emptying (Q1), intermittency (Q3), weak stream (Q5), and straining (Q6) had a tendency to increase after treatment in the old-old group.

**Table 2:** Changes in the values of the subjective and objective symptoms in each group of elderly male patients with overactive bladder

	Young-old group (*N* = 22)			Old-old group (*N* = 28)		
	0 W	12 W	*P* Value	0 W	12 W	*P* Value
OABSS						
Q1 Daytime frequency	1.1 ± 0.7	1.4 ± 0.8	0.307	1.1 ± 0.8	1.4 ± 0.5	0.148
Q2 Nighttime frequency	2.1 ± 1.0	1.5 ± 0.9	0.034	2.0 ± 0.8	1.5 ± 0.6	0.005
Q3 Urgency	2.7 ± 1.1	1.2 ± 1.1	< 0.001	2.2 ± 0.4	1.3 ± 0.6	< 0.001
Q4 Urgency incontinence	0.9 ± 1.3	0.3 ± 0.6	0.021	0.2 ± 0.4	0.3 ± 0.4	0.686
Total score	6.5 ± 2.7	4.4 ± 1.6	0.004	5.6 ± 1.3	4.2 ± 1.2	< 0.001
IPSS						
Q1 Incomplete emptying	1.2 ± 1.4	1.2 ± 1.3	1.000	1.2 ± 1.0	1.4 ± 1.2	0.093
Q2 Frequency	3.0 ± 1.1	2.0 ± 1.0	0.001	3.1 ± 0.7	2.1 ± 0.9	< 0.001
Q3 Intermittency	1.4 ± 1.3	1.2 ± 1.3	0.205	1.0 ± 1.0	1.1 ± 1.2	0.529
Q4 Urgency	2.8 ± 0.8	1.7 ± 0.9	< 0.001	2.6 ± 0.8	1.5 ± 1.0	< 0.001
Q5 Weak stream	2.2 ± 1.1	2.0 ± 1.2	0.142	2.2 ± 1.0	2.3 ± 1.0	0.689
Q6 Straining	2.0 ± 1.5	1.7 ± 1.4	0.110	1.3 ± 0.8	1.4 ± 0.9	0.529
Q7 Nocturia	3.3 ± 0.8	2.3 ± 1.4	0.001	2.9 ± 0.8	1.9 ± 0.9	< 0.001
Total score	16.0 ± 5.1	12.0 ± 5.3	< 0.001	14.5 ± 3.3	11.7 ± 4.1	< 0.001
Storage symptoms (Q2 + Q4 + Q7)	9.1 ± 1.9	6.0 ± 2.5	< 0.001	8.7 ± 5.6	5.6 ± 2.0	< 0.001
Voiding symptoms (Q1 + Q3 + Q5 + Q6)	6.8 ± 4.4	6.0 ± 4.4	0.029	5.8 ± 2.5	6.1 ± 3.0	0.102
QOL score	4.3 ± 1.0	3.5 ± 1.4	0.006	4.1 ± 0.8	3.2 ± 1.0	< 0.001
Urodynamic study						
VV (mL)	141.0 ± 78.1	169.5 ± 74.0	0.012	134.4 ± 49.2	142.0 ± 53.4	0.133
Qmax (mL/s)	9.1 ± 3.0	9.9 ± 2.7	0.159	9.1 ± 2.2	12.0 ± 12.4	0.116
PVR (mL)	27.4 ± 41.8	22.1 ± 22.7	0.879	26.5 ± 17.3	30.4 ± 25.9	0.190

*OABSS* overactive bladder symptom score, *IPSS* international prostate symptom score, *w* week, *VV* voided volume, *Qmax* maximum flow rate, *PVR* post-void residual urine

Among the objective symptoms, VV on free uroflowmetry increased after treatment in the young-old group (*P* = 0.012) but not in the old-old group (*P* = 0.113). There were no significant changes in the Qmax and PVR after treatment in either group.

A safety analysis was performed on all patients during the clinical trial. Two patients (4 %) complained of dry mouth. One patient (2 %) complained of constipation. However, since these adverse effects were very mild, the patients did not need to stop taking mirabegron. In addition, none of the patients with hypertension experienced worsening of their blood pressure levels. Hence, all patients completed this clinical study, including all scheduled examinations during the study period.

## Discussion

To the best of our knowledge, the present study is the first to focus on the efficacy of mirabegron additional therapy in elderly male patients with OAB after monotherapy with α1-adrenergic receptor blockers. Our findings indicated that mirabegron additional therapy was effective for those whose OAB was not controlled with α1-adrenergic receptor blockers, according to the OABSS and IPSS. Our results also demonstrated that mirabegron had no suppressive effects on voiding function on uroflowmetry and PVR. In addition, regardless of the patient’s age, mirabegron add-on therapy was considered effective and safe to take orally.

In elderly patients with benign prostatic hyperplasia, an α1-adrenergic receptor blocker significantly improved LUTS, especially voiding dysfunction. However, some patients may continue to suffer from storage symptoms such as urgency and frequency. For these patients, some studies have suggested that combination therapy with an α1-adrenergic receptor blocker and anti-muscarinic drug is effective [18, 19]. However, because of the impaired physiological function of elderly people, anti-muscarinic drugs carry the risk for adverse events. In fact, there is a significant incidence of peripheral anti-muscarinic adverse events such as dry mouth, constipation, tachycardia, cognitive dysfunction, and voiding dysfunction in elderly patients [20, 21]. Voiding dysfunction, in particular, due to male LUTS, might lead to excessive residual urine, urinary tract infection, and post-renal renal failure.

Mirabegron, a β3-adrenergic receptor agonist, is a new type of agent for OAB, with a reported adverse effect rate almost as low as that of a placebo [15, 22]. However, recently, concerns have been raised regarding the use of mirabegron to treat OAB patients with severe hypertension (systolic blood pressure ≥ 180 mmHg and/or diastolic blood pressure ≥ 110 mmHg) [23]. In addition, no studies have targeted elderly male patients 65 years old and over, who are more prone to experiencing complications than younger patients.

According to the present study, mirabegron additional therapy improved storage symptoms on the OABSS and IPSS regardless of age. In addition, mirabegron additional therapy improved storage symptoms and voiding symptoms on the IPSS in the young-old patient group. Previous studies have reported that mirabegron improved the OABSS and the total and storage symptoms on the IPSS in male patients with OAB [15, 16, 24]. Moreover, Wada et al. suggested that mirabegron add-on treatment with tamsulosin improved storage symptoms and voiding symptoms [16]. However, these previous studies were complex, as they included patients of various ages and they had small sample sizes; no study has focused only on older male patients. Ichihara et al. reported that combination therapy with tamsulosin and mirabegron was effective for persistent OAB symptoms with benign prostatic obstruction after tamsulosin monotherapy [15]. They divided the patients into two groups, tamsulosin monotherapy (*N* = 38) and combination therapy with tamsulosin and mirabegron (*N* = 38). Although average age of their study cohort was very similar to that of the present study (74.5 ± 8.2 vs. 75.7 ± 7.6 years), they included male patients aged 50 years or older. Hence they did not target only elderly male LUTS patients. In addition, the number of patients receiving combination therapy with tamsulosin and mirabegron was lower in the previous study compared to ours.

Upon further stratifying our study subjects according to age, our results suggested that voiding symptoms worsened slightly after treatment in the old-old patient group, but this difference was not statistically significant. These findings indicate that the use of mirabegron in elderly patients should be carefully monitored, especially in older patients .

The objective symptoms VV and Qmax on uroflowmetry were improved after treatment in patients overall. VV was particularly improved after treatment in young-old patients. However, objective symptoms did not change significantly in the old-old patient group. A previous pressure-flow study reported that mirabegron did not affect urinary bladder contraction even if older male patients were included in the analysis [16, 25]. However, these studies indicated that mirabegron therapy possibility increased PVR, although the change was not statistically significant. In our study, we obtained similar results for PVR. The older patients’ voiding functions declined physiologically, and their initial PVR volume was higher than that of the younger patients [26]. Although many clinicians think that mirabegron is associated with fewer problems in terms of voiding function than anti-cholinergic agents, it is necessary to consider changes in both the subjective and objective symptoms after treatment with mirabegron, especially in old-old patients.

In the present study, only three patients (6 %) had mild adverse effects (2 patients, thirst; 1 patient, constipation), and all patients continued to take mirabegron during the study period. None of the patients with hypertension experienced worsening of their blood pressure level, although hypertension was well-controlled by medication or not severe in all cases in the present study. Hence, it appears that mirabegron can be used safely, and it has good tolerability. Previous studies have reported that typical adverse events such as dry mouth and constipation occurred at a similar incidence between mirabegron and placebo treatment [10, 12, 27]. The incidence of dry mouth with mirabegron, in particular, is three- to four-fold lower than that with tolterodine. As noted in a meta-analysis, anti-muscarinic drugs are associated with a 29.6 % incidence of dry mouth [27]. As dry mouth is reported to be an important factor determining persistence, the favorable tolerability profile of mirabegron may result in improved treatment adherence compared with anti-muscarinic drugs, which has important implications for patient outcome [10]. Many elderly people suffer from constipation and dry mouth; thus mirabegron may be convenient to administer in the elderly with OAB [28, 29].

The present study has several limitations, the major one being that the number of patients included was very small. The observation period was also limited to only 12 weeks. In addition, this study was open label, not placebo controlled. Moreover, patients with relatively mild voiding symptoms prior to mirabegron treatment (with IPSS subscale scores for voiding symptoms of 6.8 ± 1.4, and PVR of 27.4 ± 41.8 mL) and with low prostate volume (33.7 ± 8.6 mL) unlikely to conducting the urinary dysfunction were mainly included in this study. These factors may have contributed to the low complication rate observed in this study. However, despite these limitations, this is the first prospective study that specifically evaluated elderly male patients with OAB who were administered mirabegron additional therapy after treatment with α1-adrenergic receptor blockers. In recent years, there has been growing interest in the efficacy and safety of mirabegron for elderly patients [30]. We believe that in spite of its small sample size, this investigation contributes important information to the selection of treatment strategies in elderly patients with lower urinary tract symptoms.

## Conclusions

Our results indicate that mirabegron additional treatment is effective, safe, and tolerable therapy for persistent OAB in elderly male patients after monotherapy with α1-adrenergic receptor blockers. In addition, mirabegron additional therapy was considered effective regardless of the patient’s age.

## Abbreviations

IPSS, international prostate symptom score; LUTS, lower urinary tract symptoms; OAB, overactive bladder; OABSS, overactive bladder symptom; PVR, post-void residual urine volume; Qmax, maximum flow rate; QOL, quality of life; VV, voided volume
